# Histone Deacetylation Controls Xylem Vessel Cell Differentiation *via* Transcriptional Regulation of a Transcription Repressor Complex OFP1/4–MYB75–KNAT7–BLH6

**DOI:** 10.3389/fpls.2021.825810

**Published:** 2022-01-27

**Authors:** Risaku Hirai, Shumin Wang, Taku Demura, Misato Ohtani

**Affiliations:** ^1^Graduate School of Science and Technology, Nara Institute of Science and Technology, Ikoma, Japan; ^2^Department of Botany, University of British Columbia, Vancouver, BC, Canada; ^3^RIKEN Center for Sustainable Resource Science, Yokohama, Japan; ^4^Center for Digital Green-Innovation, Nara Institute of Science and Technology, Ikoma, Japan; ^5^Department of Integrated Biosciences, Graduate School of Frontier Sciences, The University of Tokyo, Kashiwa, Japan

**Keywords:** histone deacetylation, trichostatin A, sirtinol, VND7, xylem vessel cell

## Abstract

Xylem vessels are indispensable tissues in vascular plants that transport water and minerals. The differentiation of xylem vessel cells is characterized by secondary cell wall deposition and programmed cell death. These processes are initiated by a specific set of transcription factors, called VASCULAR-RELATED NAC-DOMAIN (VND) family proteins, through the direct and/or indirectly induction of genes required for secondary cell wall deposition and programmed cell death. In this study, we explored novel regulatory factors for xylem vessel cell differentiation in *Arabidopsis thaliana*. We tested the effects of cellular stress inducers on VND7-induced differentiation of xylem vessel cells with the *VND7–VP16–GR* system, in which VND7 activity is post-translationally induced by dexamethasone application. We established that the histone deacetylase (HDAC) inhibitors trichostatin A (TSA) and sirtinol inhibited VND7-induced xylem vessel cell differentiation. The inhibitory effects of TSA and sirtinol treatment were detected only when they were added at the same time as the dexamethasone application, suggesting that TSA and sirtinol mainly influence the early stages of xylem vessel cell differentiation. Expression analysis revealed that these HDAC inhibitors downregulated VND7-downstream genes, including both direct and indirect targets of transcriptional activation. Notably, the HDAC inhibitors upregulated the transcript levels of negative regulators of xylem vessel cells, OVATE FAMILY PROTEIN1 (OFP1), OFP4, and MYB75, which are known to form a protein complex with BEL1-LIKE HOMEODOMAIN6 (BLH6) to repress gene transcription. The KDB system, another *in vitro* induction system of ectopic xylem vessel cells, demonstrated that TSA and sirtinol also inhibited ectopic formation of xylem vessel cells, and this inhibition was partially suppressed in *knat7-1, bhl6-1, knat7-1 bhl6-1*, and quintuple *ofp1 ofp2 ofp3 ofp4 ofp5* mutants. Thus, the negative effects of HDAC inhibitors on xylem vessel cell differentiation are mediated, at least partly, by the abnormal upregulation of the transcriptional repressor complex OFP1/4–MYB75–KNAT7–BLH6. Collectively, our findings suggest that active regulation of histone deacetylation by HDACs is involved in xylem vessel cell differentiation *via* the OFP1/4–MYB75–KNAT7–BLH6 complex.

## Introduction

Xylem vessels are important tissues in vascular plants that transport water and minerals. During their differentiation, xylem vessel cells develop thickened secondary cell walls (SCWs) composed of cellulose, hemicellulose, and lignin, and eventually undergo programmed cell death (PCD), resulting in a hollow structure (Turner et al., 2007; Kamon and Ohtani, 2021). Advances in molecular biological research have revealed much about the molecular mechanisms of xylem vessel cell differentiation; a factor facilitating this research was the development of an artificial induction system for xylem vessel cell differentiation (Tan et al., 2019). In 2005, an artificial induction system using cultured Arabidopsis (*Arabidopsis thaliana*) cells was established, and genome-wide transcriptome data associated with xylem vessel cell differentiation was obtained with this induction system (Kubo et al., 2005). In this work, Kubo et al. (2005) identified the VASCULAR-RELATED NAC-DOMAIN (VND) family, a group of NAC-type transcription factors whose expression is upregulated in the early stages of xylem vessel cell differentiation (Kubo et al., 2005).

The Arabidopsis VND family includes VND1 to VND7, and the *VND* genes are expressed in developing xylem vessel cells (Kubo et al., 2005; Yamaguchi et al., 2008). Overexpression of *VND* genes induces ectopic SCW thickening and programmed cell death (Kubo et al., 2005; Yamaguchi et al., 2008, 2010a; Zhou et al., 2014; Endo et al., 2015), while the artificial suppression of VND function suppresses the differentiation of xylem vessel cells (Kubo et al., 2005; Yamaguchi et al., 2010b). These results suggest that the VND family functions as a master switch for xylem vessel cell differentiation. In 2010, multiple genome-wide transcriptome analyses using the *VND6* or *VND7* inducible system revealed the direct target factors of VND proteins (Ohashi-Ito et al., 2010; Zhong et al., 2010; Yamaguchi et al., 2011). The list of VND7 direct target genes included genes involved in SCW formation, such as SCW-specific cellulose synthase genes *CELLULOSE SYNTHASE A4* (*CesA4*) and *CesA7* (Brown et al., 2005); xylan synthase genes *IRREGULAR XYLEM8* (*IRX8*) and *IRX10* (Peña et al., 2007); and PCD-related protease genes *XYLEM CYSTEINE PEPTIDASE 1* (*XCP1*) (Funk et al., 2002; Avci et al., 2008) and *METACASPASE 9* (*MC9*) (Bollhöner et al., 2013). Moreover, the downstream region of VND7 contains multiple transcription factors, such as LOB DOMAIN-CONTAINING PROTEIN 30 (LBD30), LBD15, and LBD18 (Soyano et al., 2008; Ohashi-Ito et al., 2018) as well as MYB46, MYB83, and MYB63 (Ko et al., 2009, 2012; Zhou et al., 2009; Zhong and Ye, 2012). The LBD proteins positively regulate *VND7* expression, suggesting the existence of positive feedback regulation between VND7 and LBD (Soyano et al., 2008; Ohashi-Ito et al., 2018). MYB46 and MYB83 induce the expression of SCW-related genes such as *CesA*, which is also targeted by VND7 (Ko et al., 2012; Zhong and Ye, 2012), and form a so-called feed-forward loop with VND7 (Taylor-Teeples et al., 2015; Turco et al., 2019).

While the VND family genes positively regulate xylem vessel cell differentiation, other transcription factors inhibit xylem vessel cell differentiation, including VND-INTERACTING2 (VNI2) (Yamaguchi et al., 2010b); XYLEM NAC DOMAIN1 (XND1) (Zhao et al., 2007; Zhang et al., 2020); the homeobox transcription factors BEL1-LIKE HOMEODOMAIN6 (BLH6; Liu et al., 2014; Liu and Douglas, 2015), KNOTTED ARABIDOPSIS THALIANA3 (KNAT3), and KNAT7 (Bhargava et al., 2010; Li et al., 2011, 2012; Liu and Douglas, 2015; Wang et al., 2020); OVATE FAMILY PROTEIN1 (OFP1) and OFP4 (Li et al., 2011; Liu and Douglas, 2015); and the MYB transcription factors MYB4, MYB5, MYB7, MYB32, and MYB75 (Preston et al., 2004; Ko et al., 2009; Bhargava et al., 2010, 2013). Among these, VNI2 and XND1, which are NAC-type transcription factors, have been suggested to physically interact with VND7 to form a heterodimer that represses VND7 function (Yamaguchi et al., 2010b; Zhang et al., 2020).

The above findings suggest the existence of a complex transcriptional regulatory network consisting of VND7-based positive regulation and negative regulation by multiple classes of transcription factors (Ohashi-Ito and Fukuda, 2010; Hussey et al., 2013; Nakano et al., 2015; McCahill and Hazen, 2019; Ohtani and Demura, 2019). A gene co-expression analysis suggested that the expression and interaction patterns of these transcription factor genes, including the VND family members, could be different in the presence and absence of stresses such as salt and drought (Taylor-Teeples et al., 2015; Ohtani and Demura, 2019). Molecular genetics research also revealed that the activity of VND family proteins can be actively regulated in response to light (Tan et al., 2018) and cellular thiol conditions (Kawabe et al., 2018; Ohtani et al., 2018). Thus, it is highly possible that stress can modify the transcriptional regulatory network, especially the balance between transcriptionally positive and negative regulation modules, for proper xylem vessel cell differentiation.

To obtain clues into novel regulatory factors involved in such modification, we tested the effects of cellular stresses on xylem vessel cell differentiation initiated by VND7. We used Arabidopsis *VND7–VP16–GR* plants overexpressing a chimeric protein of VND7, a transcriptional activation domain VP16, and the glucocorticoid receptor (GR) (Yamaguchi et al., 2010a). In this system, VND7 activity is post-translationally activated by treatment with glucocorticoids, such as dexamethasone (DEX), leading to ectopic induction of xylem vessel cell differentiation in a DEX concentration-dependent manner (Yamaguchi et al., 2010a; Hirai et al., 2019; Supplementary Figure 1A). We treated the Arabidopsis *VND7–VP16–GR* seedlings with known cellular stress inducers and found that the histone deacetylase (HDAC) inhibitors trichostatin A (TSA) and sirtinol significantly inhibited ectopic xylem vessel cell differentiation. Further expression analysis demonstrated that the HDAC inhibitor treatment upregulated negative regulators of xylem vessel cell differentiation, such as OVATE FAMILY PROTEIN1 (OFP1), OFP4, and MYB75, which form a protein complex with BEL1-LIKE HOMEODOMAIN6 (BLH6) to repress gene transcription. Indeed, the *knat7* and *ofp* mutations suppressed the inhibitory effects of the HDAC inhibitor on xylem vessel cell differentiation. These results suggest that HDACs play important roles in xylem vessel cell differentiation through the regulation of a transcriptional repression complex.

## Materials and Methods

### Plant Materials and Growth Conditions

The Arabidopsis (*A. thaliana*) *VND7–VP16–GR* line (Col-0) was described in Yamaguchi et al. (2010a), and *ofp1, ofp4, knat7-1, blh6-1*, and *knat7-1 blh6-1* were reported in Wang et al. (2007), Li et al. (2011), and Liu et al. (2014). T-DNA insertion lines for *ofp2* (SALK_122550, with the insertion at the 3′UTR; Supplementary Figure 2), *ofp3* (GABI_167F01, with the insertion at the exon), and *ofp5* (SALK_203823, with the insertion at the exon) were obtained from the Arabidopsis Biological Resource Center (ABRC). Quintuple *ofp1 ofp2 ofp3 ofp4 ofp5* mutants were generated by genetic crossing, and the genotypes were confirmed by PCR. The seedlings were grown on Murashige and Skoog (MS) medium (Wako, Japan) containing 1% (w/v) sucrose (Nacalai tesque, Japan), 0.05% (w/v) MES (nacalai tesque), and 0.6% (w/v) Gellan gum (Wako), adjusted to pH 5.7 under continuous light at 22°C.

### DEX Treatment and Cellular Stress Inducer Treatment

Seven-day-old seedlings of the *VND7–VP16–GR* line were treated with 10 nM DEX (Sigma-Aldrich, St. Louis, MO, USA), as previously described in Hirai et al. (2019). Briefly, the seedlings were transferred into a 12-well plate (Corning) containing the 10 nM DEX solution and incubated for 3 d. For the treatment with cellular stress inducers (Supplementary Table 1), the inducers were added to the 10 nM DEX solution with the final concentration described in Supplementary Table 1; in addition to the reported concentration that can affect plant cell activity (Supplementary Table 1), one-tenth and 10-fold concentrations were tested for each chemical. To check the involvement of protein *S*-nitrosylation, or auxin response, in the downstream of HDAC inhibitors, the *VND7–VP16–GR* seedlings were treated by 10 nM DEX and 5 μM TSA, in the presence or absence of 500 μM 2-(4-carboxyphenyl)-4,4,5,5-tetramethylimidazoline-1-oxyl-3- oxide (cPTIO), a nitric oxide scavenger, or by 10 nM DEX, in the presence or absence of 10 μM 1-naphthaleneacetic acid (NAA), for 3 d, respectively. To examine the time window where HDAC inhibitors can influence xylem vessel cell differentiation, TSA and sirtinol were added at 0, 6, and 12 h of DEX treatment.

### KDB Treatment

KBD treatment was carried out as described previously (Tan et al., 2018, 2019). The cotyledons of 7-day-old seedlings were excised and incubated in half-strength MS liquid medium supplemented with KDB hormone mixture (50 ng/ml kinetin, 500 ng/ml 2,4-dichlorophenoxyacetic acid, and 1 μm brassinolide) at 22°C under continuous light for 4 d.

### Microscopy Observation

The SCW deposition ratio in the *VND7–VP16–GR* leaves was determined according to the method in Hirai et al. (2019). Cotyledon samples were fixed, stained with propidium iodide (PI), and mounted using TOMEI-I (Hasegawa et al., 2016; Hirai et al., 2019). These samples were observed with a FV-10i confocal microscope (Olympus). The confocal images were processed using ImageJ (https://imagej.nih.gov/ij/index.html) with the plugin MosaicJ (Thévenaz and Unser, 2007) to obtain maximum intensity projection images of whole cotyledons. We measured the area with SCW-signals manually and calculated the ratio of SCW-positive cell regions.

To observe KDB-treated cotyledons, the cotyledon samples were fixed with a mixture of 10% (v/v) acetic acid and 90% (v/v) ethanol. The samples were then hydrated with a 90, 70, 50, and 30% (v/v) ethanol series for 20 min each and then transferred into distilled water. Finally, the samples were mounted in clearing solution (chloral hydrate:water:glycerol 8:1:2 [w/v/v]). Images were taken using a light microscope (BX53; Olympus) equipped with differential interference contrast (DIC) optics and a digital camera (DP72; Olympus).

### Reverse Transcription Quantitative PCR Analysis

Reverse transcription quantitative Quantitative RT-PCR analysis was performed as described in Yamaguchi et al. (2011) and Hirai et al. (2019). The seedlings were collected at 0, 6, 12, 18, 24, 48, and 72 h after DEX treatment and then ground in liquid nitrogen with a TissueLyser II (Qiagen). Total RNA was isolated using an RNeasy Plant Mini Kit (Qiagen) according to the manufacturer's instructions. cDNA synthesis was performed with 1 μg of total RNA using a Transcriptor First-Strand cDNA Synthesis Kit (Roche). Quantitative PCR was carried out with LightCycler 480 DNA SYBR Green (Roche). The expression levels of tested genes were normalized with that of *UBQ10*. The primers used in this study are shown in Supplementary Table 2.

### Statistical Analysis

For the statistical analysis of ratio of SCW positive regions and gene expression levels, we performed Student's *t*-test between the mock-treated control and the samples treated with TSA or sirtinol. In the case of multiple comparison test, we performed Tukey's test (Tukey HSD, honestly significant difference). All tests were performed with Microsoft Excel software (ver 16.55, Microsoft) or in R (https://www.R-project.org).

## Results and Discussion

### HDAC Inhibitors Inhibit VND7-Induced Xylem Vessel Cell Differentiation

To identify novel factors affecting VND7-induced xylem vessel cell differentiation, we examined the effects of known cellular stress inducers (50 chemicals; Supplementary Table 1) on ectopic differentiation of xylem vessel cells in *VND7–VP16–GR* (Supplementary Figure 1A). First, we screened the cellular stress inducers by determining if they inhibited bleaching of cotyledons, which is considered to reflect PCD progression (Yamaguchi et al., 2010a; Hirai et al., 2019; Supplementary Figure 1B). After the first screening, 12 chemicals were found to inhibit the bleaching of cotyledons (Supplementary Figure 1B). As a second screening, the treated cotyledons were examined for SCW deposition (Supplementary Figure 1B). Of the 12 chemicals, five (i.e., citrate acid, oxidized glutathione (GSSG, glutathione-S-S-glutathione), reduced glutathione (GSH, glutathione-SH), trichostatin A (TSA), and sirtinol), reduced the ratio of SCW deposition significantly (Supplementary Figure 3).

**Figure 1:**
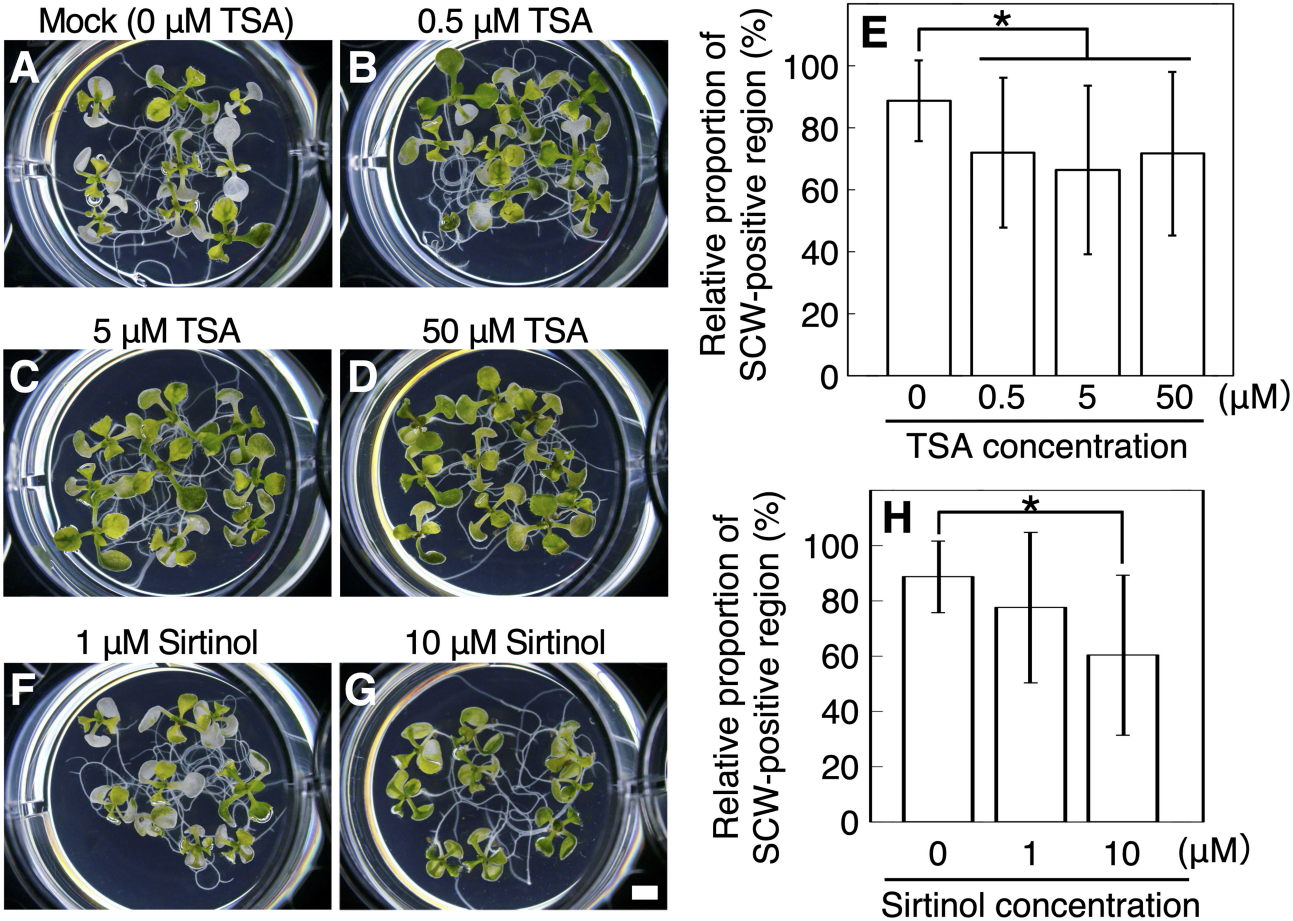
Histone deacetylase inhibitors inhibit VND7-based xylem vessel cell differentiation. **(A–E)** Seven-day-old *VND7–VP16–GR* seedlings were soaked in the distilled water with 10 nM dexamethasone (DEX) and 0 μM **(A)**, 0.5 μM **(B)**, 5 μM **(C)**, or 50 μM **(D)** trichostatin A (TSA) for 3 d. **(F–H)** The *VND7–VP16–GR* seedlings were treated with 10 nM DEX and 1 μM **(F)** or 10 μM **(G)** sirtinol, and incubated for 3 d. **(E,H)** Ectopic deposition of secondary cell walls (SCWs) in cotyledons was observed with a confocal microscope after propidium iodide staining, and the SCW-positive regions were measured in each cotyledon. The relative proportions of SCW-positive cell regions were calculated manually using an image analysis performed in ImageJ. Results are shown as means ± SD (*n* > 10). Asterisks indicate statistically significant differences compared with the mock control (Student's *t*-test, *p* < 0.05). Bar, 1 mm.

The *S*-nitrosylation of VND7 is important for the regulation of VND7 transcriptional activity (Kawabe et al., 2018; Ohtani et al., 2018). GSSG and GSH can affect cellular thiol environments, possibly leading to the disturbance of protein *S*-nitrosylation regulation (Meyer and Hell, 2005). Thus, the inhibitory effects of GSSG and GSH on xylem vessel cell differentiation in *VND7–VP16–GR* may reflect such functional disruption of VND7 by the disturbance of cellular thiol homeostasis. Interestingly, TSA and sirtinol, which are histone deacetylase (HDAC) inhibitors (Grozinger et al., 2001; Chang and Pikaard, 2005; Bourque et al., 2011; Liu et al., 2017; Mengel et al., 2017; Ueda et al., 2017), were among the five chemicals that substantially reduced the ratio of SCW deposition (Supplementary Figure 3). TSA and sirtinol are known to inhibit class I/II and class III HDACs, respectively (Yoshida and Horinouchi, 1999; Grozinger et al., 2001). Our detailed analysis showed that more than 0.5 μM TSA and 10 μM sirtinol significantly inhibited the bleaching of seedlings (Figures 1A–G) as well as SCW deposition in cotyledons (Figures 1E,H). These observations indicate that HDAC activity is important for VND7-induced xylem vessel cell differentiation.

TSA can also promote protein *S*-nitrosylation (Mengel et al., 2017), and sirtinol can induce auxin response (Zhao et al., 2003). Therefore, we tested whether the negative effect of TSA and sirtinol on xylem vessel cell differentiation depends on protein *S*-nitrosylation or auxin response. For this, we co-treated *VND7–VP16–GR* seedlings with the nitric oxide scavenger 2-(4-carboxyphenyl)-4,4,5,5-tetramethylimidazoline-1-oxyl-3-oxide (cPTIO), which inhibits protein *S*-nitrosylation (Mengel et al., 2017; Ageeva-Kieferle et al., 2021), or with synthetic auxin 1-naphthaleneacetic acid (NAA). The inhibition of xylem vessel cell differentiation by TSA was not affected by the additional application of cPTIO (Supplementary Figure 4), and NAA did not inhibit the differentiation (Supplementary Figure 5), suggesting that the inhibitory effect on VND7-induced xylem vessel cell differentiation was not due to protein *S*-nitrosylation nor auxin response.

### HDAC Inhibitors Disturb the Early Stages of Xylem Vessel Cell Differentiation

**Figure 2:**
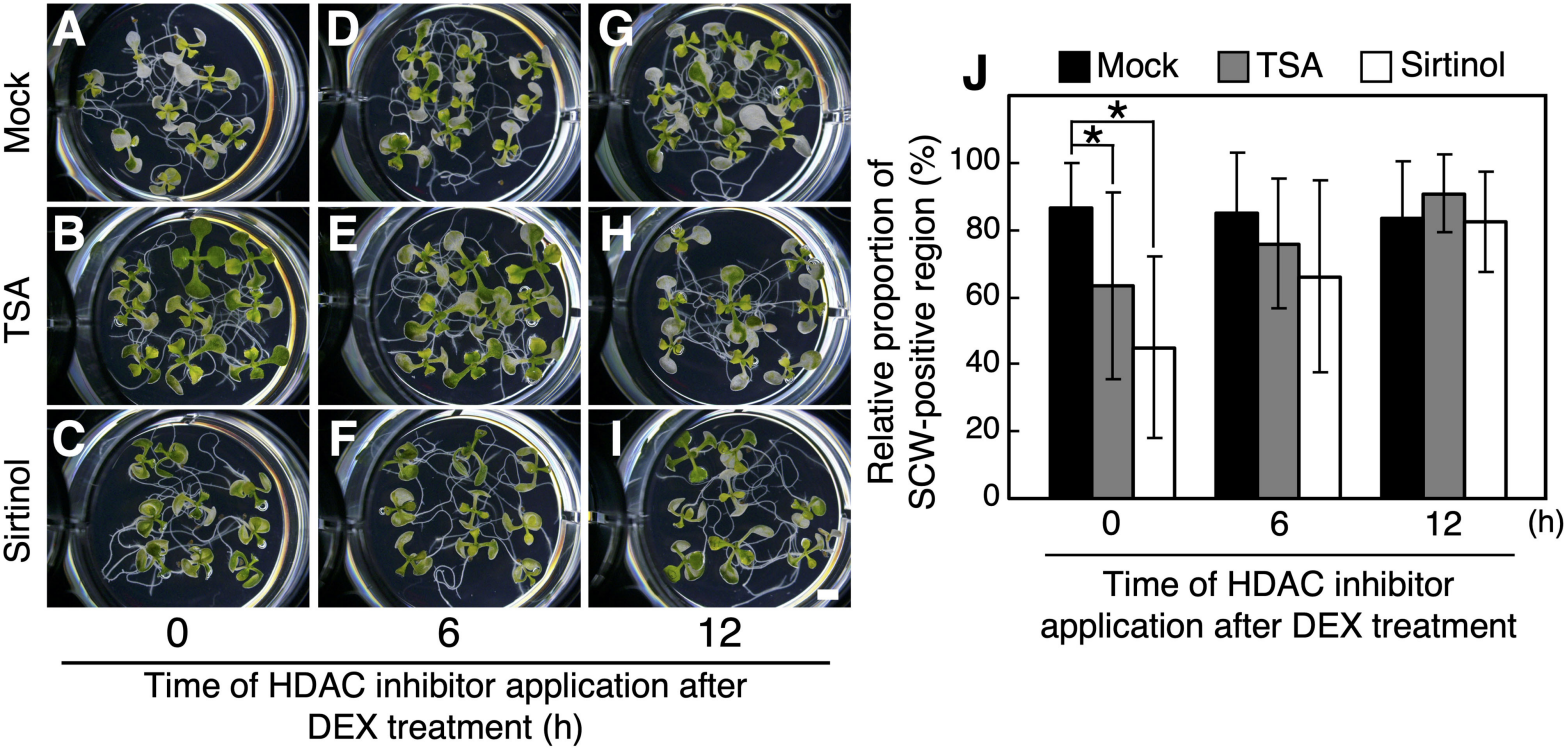
Effects of application time of histone deacetylase inhibitors on VND7-based xylem vessel cell differentiation. Seven-day-old *VND7–VP16–GR* seedlings were treated with 10 nM dexamethasone (DEX) without any inhibitors **(A–C)**, with 5 μM trichostatin A (TSA) **(D–F)**, or with 10 μM sirtinol **(G–I)** for 3 d. TSA or sirtinol was added at 0 h **(A,D,G)**, 6 h **(B,E,H)**, and 12 h of DEX treatment. **(J)** Ectopic deposition of secondary cell walls (SCWs) in cotyledons was observed with a confocal microscope after propidium iodide staining, and the SCW-positive regions were measured in each cotyledon. The relative proportions of SCW-positive cell regions were calculated manually using an image analysis performed in ImageJ. Results are shown as means ± SD (*n* > 10). Asterisks indicate statistically significant differences compare with the mock control (Student's *t*-test, *p* < 0.05). Bar, 1 mm.

Next, to examine the effect of application timing of HDAC inhibitors on xylem vessel cell differentiation, TSA and sirtinol were added to the *VND7–VP16–GR* seedlings at 0, 6, and 12 h of DEX treatment. We found a significant reduction in the degree of bleaching and SCW deposition when TSA or sirtinol were added at 0 h of DEX treatment (Figures 2A–C,J). By contrast, when we added TSA or sirtinol at 6 or 12 h of DEX treatment, no significant difference was detected in the degree of bleaching and SCW deposition, although the addition of HDAC inhibitors at 6 h of DEX treatment slightly decreased the degree of SCW deposition (Figures 2D–J). This clearly demonstrated that HDAC inhibition affected the molecular processes at the early stages of xylem vessel cell differentiation, which occur up to 6 h after DEX treatment.

### HDAC Inhibitors Suppress the Upregulation of VND7-Downstream Genes, Especially at Early Stages of Xylem Vessel Cell Differentiation

**Figure 3:**
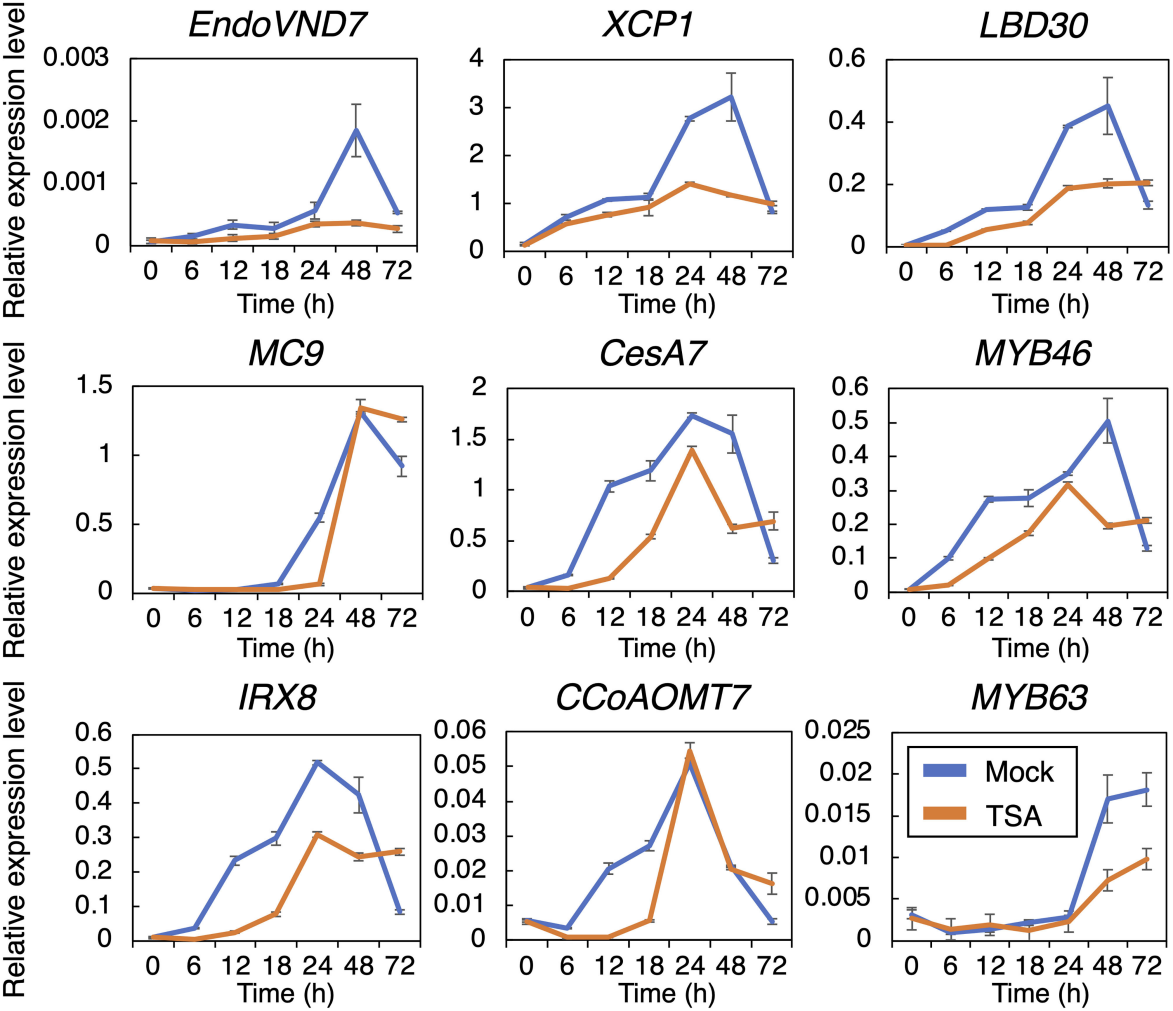
Reverse transcription quantitative PCR analysis of the genes downstream of VND7 in *VND7–VP16–GR* seedlings treated with dexamethasone (DEX) and trichostatin A (TSA). Seven-day-old *VND7–VP16–GR* seedlings were treated with 10 nM DEX and 5 μM TSA and sampled after 0, 6, 12, 18, 24, 48, and 72 h. The expression levels of the genes downstream of VND7 were normalized to the expression level of the internal control *UBIQUITIN10*. Results are shown as means ± SD (*n* = 3).

Our observations indicated that the HDAC inhibitors inhibited both SCW deposition and PCD progression (Figures 1, 2). Therefore, we further determined the effects of TSA and sirtinol on the expression of VND7 downstream genes (Figure 3 and Supplementary Figure 6). Seven-day-old *VND7–VP16–GR* seedlings were treated with the 10 nM DEX solution with or without TSA or sirtinol. Quantitative RT-PCR analysis was performed for VND7 downstream genes, such as the PCD-related genes *XCP1* and *MC9*; transcription factor genes *LBD30, MYB46*, and *MYB63*; cellulose and xylan biosynthesis genes *CESA7/IRX3* and *IRX8*, respectively; and a lignin biosynthesis gene *CAFFEOYL COENZYME A ESTER O-METHYLTRANSFERASE7* (*CCoAOMT7*). In addition, we examined the expression of endogenous *VND7*. The upregulation of these VND7 downstream genes was basically repressed by treatment with TSA or sirtinol (Figure 3 and Supplementary Figure 6). Gene upregulation that occurred within 12 h of DEX treatment was strongly inhibited (Figure 3 and Supplementary Figure 6), in accordance with the results shown in Figure 2. Collectively, these results indicate that the HDAC inhibitors affected the molecular events at the very early stages of xylem vessel cell differentiation, possibly inhibiting VND7 activity itself or processes close to the transactivation of gene expression by VND7.

**Figure 4:**
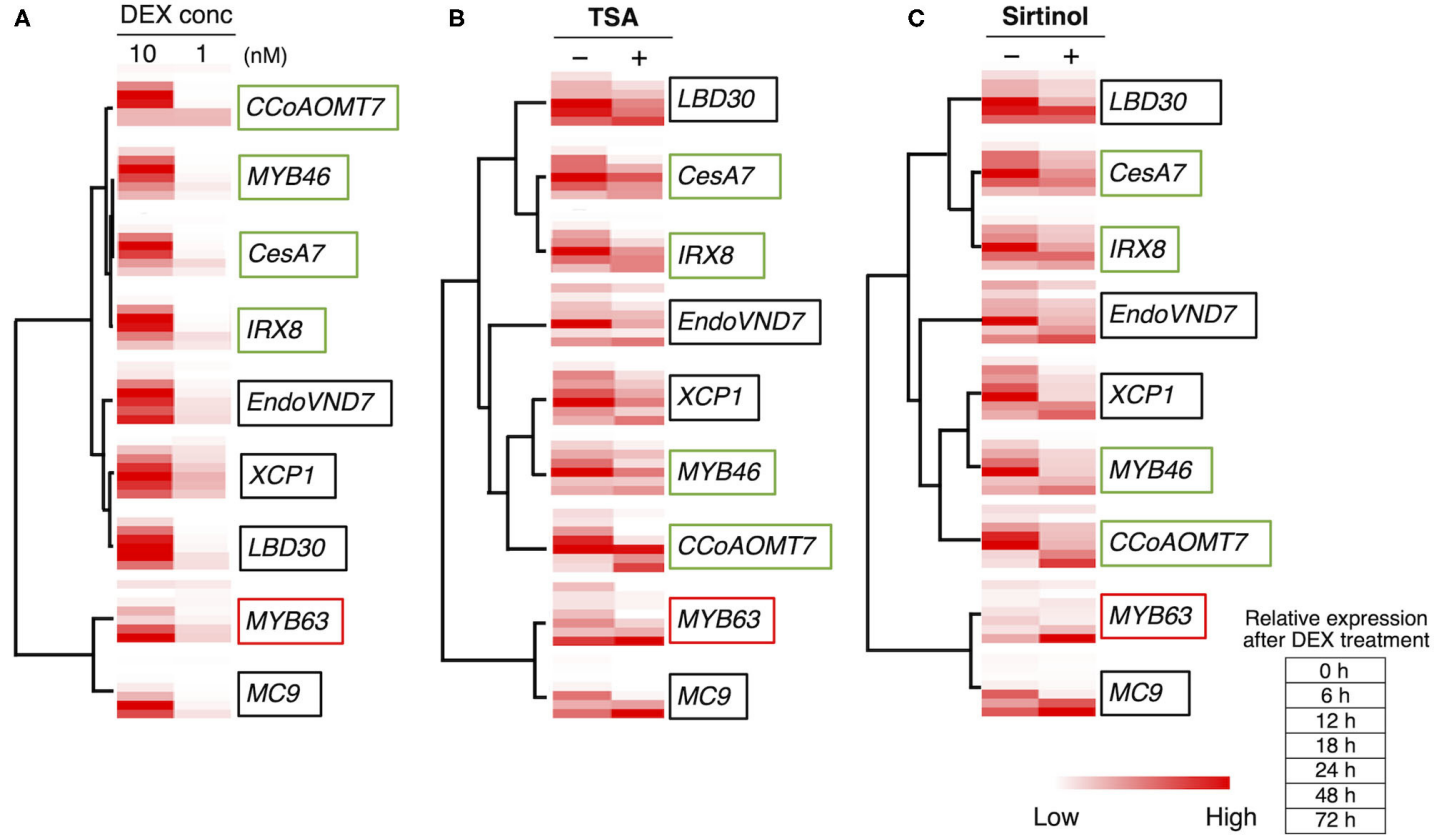
Hierarchical clustering analysis of the expression patterns of the genes downstream of VND7. The hierarchical clustering analysis was performed using the normalized quantitative RT-PCR data presented in Hirai et al. (2019) **(A)**, in Figure 3 **(B)**, and in Supplementary Figure 3 **(C)**. Each column shows the relative expression level of each gene at each time point of DEX treatment by color shading.

Previously, a DEX concentration-dependent reduction in xylem vessel cell differentiation was observed in *VND7–VP16–GR* seedlings (Hirai et al., 2019). This indicates that the degree of VND7 activity, that is, the transactivation activity of downstream genes, is a crucial factor determining the progression of xylem vessel cell differentiation (Hirai et al., 2019). To clarify how TSA and sirtinol affect VND7-based transcriptional regulation for xylem vessel cell differentiation, we performed a hierarchical clustering analysis of VND7 downstream genes based on their expression patterns (shown in Figure 3 and Supplementary Figure 6). The published data showed that *LBD30, MYB46, XCP1*, and *MC9* are direct targets of VND7 (Ohashi-Ito et al., 2010; Zhong et al., 2010; Yamaguchi et al., 2011), whereas *MYB63, CESA7, IRX8*, and *CCoAOMT7* are direct targets of MYB46 (Ko et al., 2009; Kim et al., 2012; Zhong and Ye, 2012). The genes could be separated into three groups based on their DEX concentration-dependent expression patterns. First group contained *MYB46* and its direct targets (*CESA7, IRX8*, and *CCoAOMT7*), second group contained *VND7* and two of its direct targets (*LBD30* and *XCP1*), and third one was *MYB63* (Figure 4A), reflecting the transcriptional hierarchy of VND7 and MYB46 (Figure 4A; Hirai et al., 2019). However, the expression patterns in TSA- and sirtinol-treated samples differently grouped these genes (Figures 4B,C). This suggests that the HDAC inhibitors do not simply repress VND7 activity as the decrease in DEX concentration does, but possibly change the regulatory relationship between the transcription factor and targets. Notably, the cluster structures were similar between the TSA- and sirtinol-treated samples (Figures 4B,C). Therefore, both class I/II and class III HDAC activities might be important for maintaining the transcriptional hierarchy of VND7 and MYB46 for the proper progression of xylem vessel cell differentiation.

### HDAC Inhibitors Inhibit Xylem Vessel Cell Differentiation Through the Upregulation of the OFP1/4–MYB75–KNAT7–BLH6 Transcriptional Repression Complex

**Figure 5:**
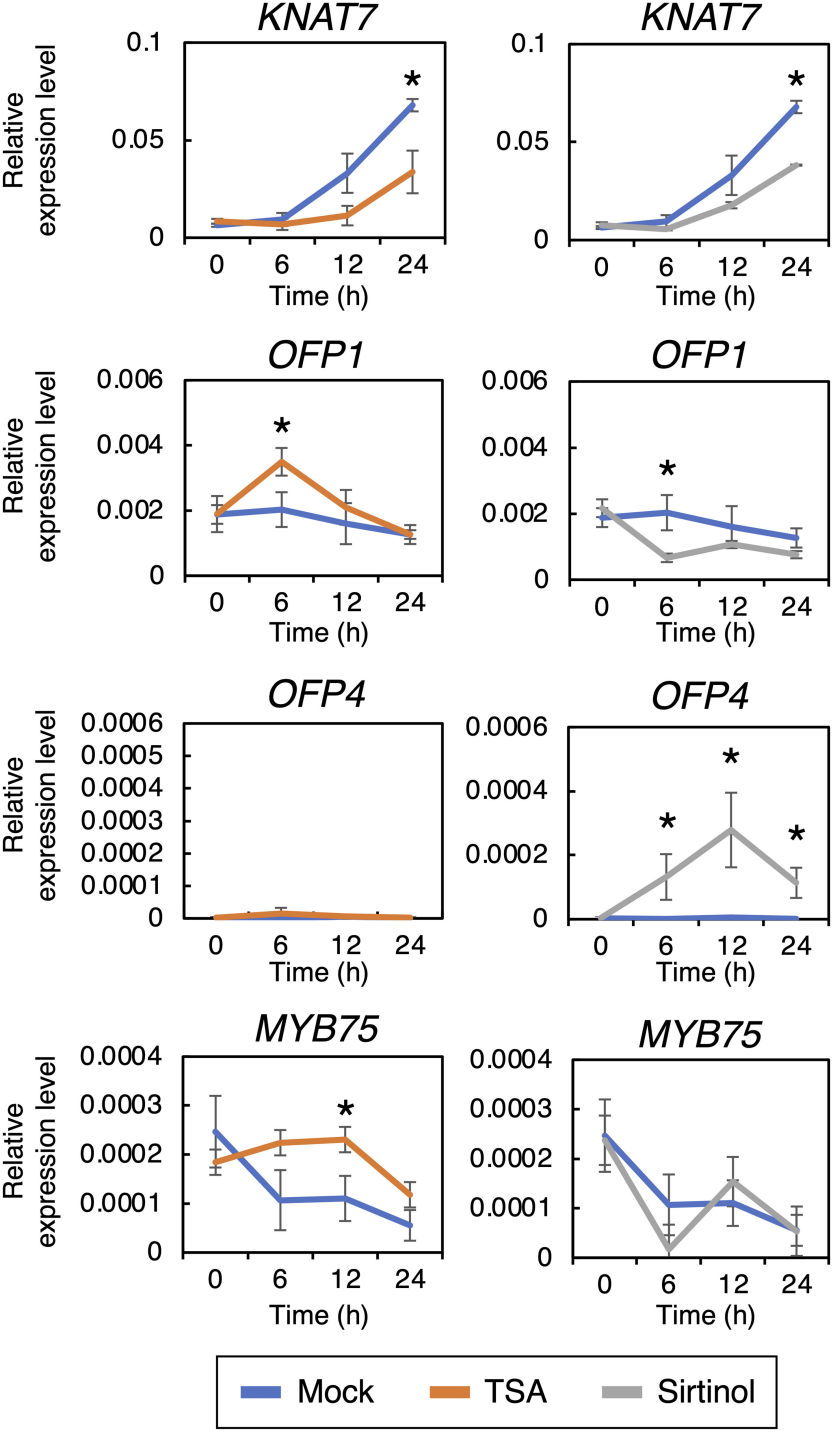
Reverse transcription quantitative PCR analysis of genes encoding transcription factors known to negatively regulate xylem vessel cell differentiation. Seven-day-old *VND7–VP16–GR* seedlings were treated with 10 nM dexamethasone (DEX) and 5 μM trichostatin A TSA or 10 μM sirtinol and sampled after 0, 6, 12, 18, and 24 h. The transcript levels of the genes downstream of VND7 were quantified by reverse transcription quantitative PCR and normalized to the expression level of the internal control *UBIQUITIN10*. Results are shown as means ± SD (*n* = 3). Asterisks indicate statistically significant differences compared with the mock control (Student's *t*-test, *p* < 0.05).

The inhibition of HDACs should lead to histone hyperacetylation, resulting in an increase in gene expression. Therefore, we hypothesized that the HDAC inhibitors would upregulate the expression of negative regulators of xylem vessel cell differentiation. The expression patterns of genes encoding 11 well-known transcription factors functioning as negative regulators of xylem vessel cell differentiation, namely *VNI2, KNAT7, XND1, BLH6, OFP1, OFP4, MYB4, MYB5, MYB7, MYB32*, and *MYB75*, were assessed (Figure 5 and Supplementary Figures 7, 8). Among these negative regulators, *KNAT7, OFP1, OFP4*, and *MYB75* were differentially expressed under the HDAC inhibitor treatment; the TSA treatment upregulated *OFP1* and *MYB75*, whereas sirtinol increased *OFP4* expression, within 12 h of DEX treatment (Figure 5). Both inhibitors also decreased the expression levels of *KNAT7* after 24 h of DEX treatment. Since the HDAC inhibitors would disturb early stages of xylem vessel cell differentiation, i.e., within 6 h after the DEX treatment (Figure 2), the inhibitory effects of HDAC inhibitors on xylem vessel cell differentiation can be attributed to the upregulation of *OPF1, OFP4*, and *MYB75* at the early stages of xylem vessel cell differentiation. OPF1, OFP4, and MYB75 form a transcriptional repression complex with KNAT7 and BLH6 to repress the expression of genes required for xylem cell differentiation (Bhargava et al., 2010; Li et al., 2011, 2012; Liu et al., 2014; Liu and Douglas, 2015; Wang et al., 2020). Thus, these findings strongly suggest that the HDAC inhibitors inhibit xylem cell differentiation through an increase in the activity of the OFP1/4–MYB75–KNAT7–BLH6 transcriptional repression complex.

**Figure 6:**
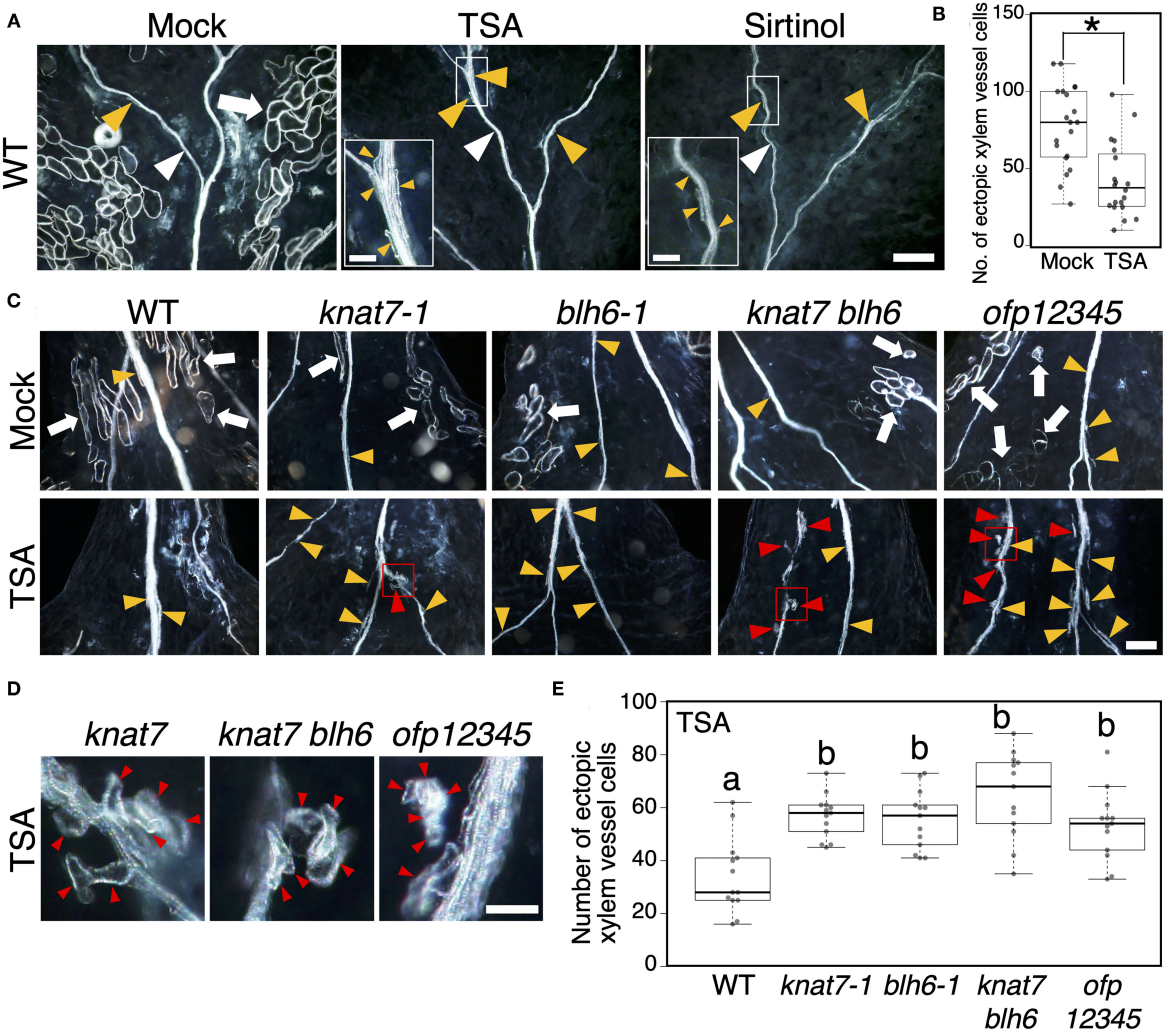
Histone deacetylase inhibitors disturb ectopic xylem vessel cell differentiation in the KDB system. **(A)** Cotyledons were cut from seven-day-old wild-type seedlings, and ectopic xylem vessel cell differentiation was induced by phytohormonal treatment (the KDB system: Tan et al., 2018), with dimethyl sulfoxide(DMSO) (mock control), 5 μM trichostatin A (TSA), or 10 μM sirtinol, for 5 d. An enlarged view of the area enclosed by the white square is shown in the inset. **(B)** Box plot of the number of ectopic xylem vessel cells per cotyledon (*n* = 20). Asterisks indicate statistically significant differences compared with the mock control (Student's *t*-test, *p* < 0.0001). **(C)** Cotyledons were cut from seven-day-old of wild-type, *knat7-1, blh6-1, knat7 blh6*, and *ofp1 ofp2 ofp3 ofp4 ofp5* (*ofp12345*) seedlings, and ectopic xylem vessel cell differentiation was induced by phytohormonal treatment (the KDB system: Tan et al., 2018) with DMSO (mock) or 5 μM TSA. **(D)** Close-up views of the area enclosed by the red square in **(C)**. **(E)** Box plot of the number of ectopic xylem vessel cells per cotyledon under the TSA treatment (*n* = 13). Different characters indicate statistically significant differences (Tukey's test, *p* < 0.05). White triangles, endogenous xylem vessels; white arrows, ectopic xylem vessel cells transdifferentiated from mesophyll cells; yellow triangles, ectopic xylem vessel cells originating from vascular cells around endogenous xylem vessels; red triangles, ectopic xylem vessel cells located near endogenous xylem vessels originating from non-vascular cells. In box plots, center lines show the medians; box limits indicate the 25th and 75th percentiles; whiskers extend 1.5 times the interquartile range from the 25th and 75th percentiles, outliers are represented by dots [*n* = 20 in **(B)**, and 13 in **(E)**]. Bars, 200 μm **(A,C)** and 50 μM [insets of **(A,D)**].

To test this possibility, we performed a mutant analysis with *knat7-1, blh6-1, knat7-1 blh6-1*, and quintuple *ofp1 ofp2 ofp3 ofp4 ofp5* mutants with a different induction system for xylem cell differentiation, the KDB system (Tan et al., 2018; Figure 6). *OFP1, OFP2, OFP3, OFP4*, and *OFP5* are close homologs in Arabidopsis. Therefore, as expected, single *ofp* mutants did not show any obvious phenotypic differences in vascular bundles compared with the wild type (Supplementary Figure 9). Thus, we newly established the quintuple *ofp1 ofp2 ofp3 ofp4 ofp5* mutants. In the KDB system, phytohormone treatment can induce ectopic xylem vessel cells (Tan et al., 2018, 2019). In the wild type, we recognized two types of ectopic xylem vessel cells: ectopic xylem vessel cells transdifferentiated from mesophyll cells (indicated by white arrows in Figure 6A) and ectopic xylem vessel cells around endogenous xylem vessels, probably originating from vascular cells (indicated by yellow triangles in Figure 6A). Interestingly, TSA and sirtinol strongly inhibited the transdifferentiation of ectopic xylem vessel cells from mesophyll cells (Figure 6A). HDAC inhibitor treatment significantly reduced the total number of ectopic xylem vessel cells (Figure 6B). We then checked the *knat7-1, blh6-1, knat7-1 blh6-1*, and *ofp1 ofp2 ofp3 ofp4 ofp5* mutants for ectopic xylem vessel cell differentiation in the presence or absence of TSA treatment (Figures 6C–E). Under the mock-treated condition, none of the mutants differed significantly from the wild type with respect to their efficiency of ectopic xylem vessel cell differentiation (Figure 6C and Supplementary Figure 10). However, TSA treatment significantly increased the number of ectopic xylem vessel cells in the mutants compared with the wild type (Figure 6E), clearly indicating the involvement of KNAT7, BLH6, and OFPs in the negative effects of TSA on ectopic xylem vessel cell differentiation, as expected. However, any tested mutations could not recover the transdifferentiation of mesophyll cells into xylem vessel cells after the TSA treatment (Figure 6C). Instead, we recognized the mutant-specific types of ectopic xylem vessel cells, which were located near endogenous xylem vessels, but their origin did not appear to be vascular cells based on their cell shapes (indicated by red triangles in Figures 6C,D). These observations collectively suggest that the OFP1/4–MYB75–KNAT7–BLH6 transcriptional repression complex is involved in the inhibition of xylem vessel cell differentiation in the cells near vascular tissues, but not in mesophyll cells, under treatment with HDAC inhibitors.

It has been shown that the transdifferentiation of mesophyll cells into xylem vessel cells is required cell dedifferentiation and vascular stem cell formation (Kondo et al., 2016; Tan et al., 2018; Furuya et al., 2021), which should contain multiple factors regulated by HDACs. In contrast, the cells near vascular tissues, such as vascular bundle sheath cells, are known to be differentiated into xylem vessels in response to stresses (Reusche et al., 2012), suggesting that they should keep certain levels of competency for xylem vessel cell differentiation originally. Therefore, the HDAC regulatory targets would be limited to the OFP1/4–MYB75–KNAT7–BLH6 complex in the cells near vascular tissues. Moreover, considering the fact that the OFP1/4–MYB75–KNAT7–BLH6 complex can repress the gene transcription by transactivation domain such as VP16 in the transient expression assay (Li et al., 2011, 2012; Liu et al., 2014; Liu and Douglas, 2015; Wang et al., 2020), it is highly possible that this complex would directly repress the transactivation of genes for xylem vessel cell differentiation by VND7, MYB46 and/or MYB83, which are transcriptional activators. Further analysis will reveal the details of molecular mechanisms for the OFP1/4–MYB75–KNAT7–BLH6 complex-based inhibition of xylem vessel cell differentiation.

## Conclusion and Perspectives

In the current work, we demonstrated the roles of histone deacetylation in regulating the OFP1/4–MYB75–KNAT7–BLH6 transcriptional repression complex (Li et al., 2011, 2012; Liu et al., 2014; Liu and Douglas, 2015; Wang et al., 2020) during xylem vessel cell differentiation (Supplementary Figure 11). The Arabidopsis genome harbors 22 genes that encode HDAC proteins, which are classified into three groups, REDUCED POTASSIUM DEFICIENCY 3 (RPD3)-like HDACs (class I; 16 genes), HD-tuins (class II; 4 genes), and sirtuins (class III; 2 genes) (Hollender and Liu, 2008). The knockout mutant of *HDT1*, one of HD-tuins type HDAC genes, produced the decreased size of xylem vessels with enhanced SCW thickness (Zhang et al., 2019), suggesting a relationship between xylem vessel cell differentiation and HD-tuins type HDACs. In addition, *OFP1* and *MYB75* expression has been reported to be increased in HDAC mutants; *OFP1* and *MYB75* are upregulated in *srt1 srt2* and in *hda19*, respectively (Zhang et al., 2018; Ning et al., 2019). Moreover, the histone acetylation level was increased at the *OFP1* gene locus in *hda6* and in the *MYB75* gene locus in *hda19* (Ning et al., 2019; Ageeva-Kieferle et al., 2021), suggesting that *OFP1* and *MYB75* are targets of histone acetylation-based active regulation of gene expression. Further analysis of the contribution of HDA proteins to the regulation of *OFP1* and *MYB75* expression will provide insight into how the OFP1/4–MYB75–KNAT7–BLH6 transcriptional repression complex affects xylem vessel cell differentiation.

Previous studies have shown that xylem vessel cell differentiation is affected by a variety of environmental stresses, such as wounding (Jacobs, 1952; Comer, 1978), salt stress (Hilal et al., 1998; Taylor-Teeples et al., 2015), bacterial infection (Reusche et al., 2012), and the light environment (Tan et al., 2018). Moreover, glutathione (Henmi et al., 2001, 2005) and nitric oxide (NO) (Kawabe et al., 2018; Ohtani et al., 2018) have been reported to affect xylem vessel cell differentiation. In particular, VND7 activity is affected by *S*-nitrosoglutathione (GSNO) application and VND7 can be *S*-nitrosylated to regulate the transcriptional activity of VND7 (Kawabe et al., 2018; Ohtani et al., 2018). Accordingly, we identified GSSG and GSH, important regulators of cellular thiol homeostasis, as inhibitors of VND7-based xylem vessel cell differentiation (Supplementary Figure 3). Recently, it has been reported that HDA6 is *S*-nitrosylated in response to NO *via* GSNO metabolism (Ageeva-Kieferle et al., 2021). This observation strongly suggests that HDAC, a crucial epigenomic regulator linking stress responses and gene expression (Mengel et al., 2017; Song et al., 2017; Ueda et al., 2017), is a key modulator of VND7-based transcriptional switching for xylem vessel cell differentiation (Supplementary Figure 11).

In summary, we suggest a novel environmental response strategy in plants, in which xylem vessel cell differentiation is regulated to match xylem vessel activities with environmental conditions. HDACs are a key part of this process, regulating histone deacetylation at the *OFP1/4* and *MYB75* gene loci (Supplementary Figure 9). The involvement of histone methylation in xylem cell formation was reported in *Eucalyptus grandis* (Hussey et al., 2015, 2017) and in Arabidopsis (Wang et al., 2021). In the case of Arabidopsis stem development, Wang et al. (2021) demonstrated that ARABIDOPSIS HOMOLOG of TRITHORAX1 (ATX1), a H3K4-histone methyltransferase, directly regulates the H3k4me3 methylation levels of the gene loci for *SECONDARY WALL-ASSOCIATED NAC DOMAIN PROTEIN1* (*SND1*) and *NAC SECONDARY WALL THICKENING PROMOTING FACTOR1* (*NST1*), which are critical transcriptional factors for fiber cell differentiation (Zhong et al., 2006; Mitsuda et al., 2007). *SND1* and *NST1* are included in the sister group to VND family proteins (Kubo et al., 2005; Zhong et al., 2006; Mitsuda et al., 2007; Nakano et al., 2015). Therefore, we postulate that both histone methylation and histone acetylation are important for the modification of the NAC–MYB-based transcriptional network for xylem cell formation (Nakano et al., 2015; Ohtani and Demura, 2019). Future analysis will clarify the details of histone modification-based regulation of xylem vessel cell differentiation.

## Data Availability Statement

The original contributions presented in the study are included in the article/Supplementary Material, further inquiries can be directed to the corresponding author.

## Author Contributions

RH, TD, and MO designed the experiments. RH performed the screening of chemicals and gene expression analysis. RH, SW, and MO performed the mutant analysis. RH, SW, TD, and MO wrote the manuscript. All authors contributed to the article and approved the submitted version.

## Funding

This work was in part supported by the RIKEN Center for Sustainable Sciences, MEXT KAKENHI (JP18H05484 and JP18H05489 to MO and TD; JP20H05405 and JP21H05652 to MO), JSPS KAKENHI (JP20H03271 to MO and JP18H02466 to TD), ERATO JST (JPMJER1602 to MO), the Toray Science Foundation (No. 19-6002 to MO), the Naito Foundation (to MO), and the Asahi Glass Foundation (to MO).

## Conflict of Interest

The authors declare that the research was conducted in the absence of any commercial or financial relationships that could be construed as a potential conflict of interest.

## Publisher's Note

All claims expressed in this article are solely those of the authors and do not necessarily represent those of their affiliated organizations, or those of the publisher, the editors and the reviewers. Any product that may be evaluated in this article, or claim that may be made by its manufacturer, is not guaranteed or endorsed by the publisher.
