# Supplementary material for: Histone Deacetylation Controls Xylem Vessel Cell Differentiation via Transcriptional Regulation of a Transcription Repressor Complex OFP1/4–MYB75–KNAT7–BLH6
Source: Front Plant Sci. 2022 Jan 27;12:825810. doi: 10.3389/fpls.2021.825810 (PMC8829346; doi:10.3389/fpls.2021.825810)
Supplement: Supplementary file 1 [file Data_Sheet_1.PDF]

Supplementary Material

1 Supplementary Figure and Table

1.1 Supplementary Figure

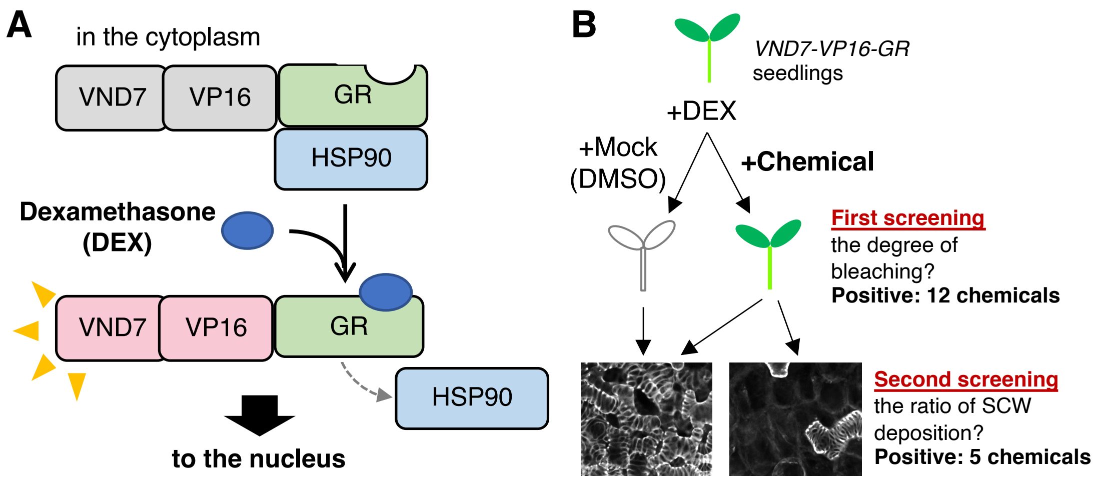

Supplementary Figure 1. Screening strategy for cellular stress inducers affecting xylem vessel cell differentiation. (A) Schematic showing the mode of action of the *VND7–VP16–GR* system. The chimeric VND7–VP16–GR protein is localized in the cytoplasm in the absence of a glucocorticoid, since the glucocorticoid receptor (GR) interacts with heat shock protein 90 (HSP90) to prevent its entry into the nucleus. In the presence of glucocorticoid molecules, such as the synthetic glucocorticoid dexamethasone (DEX), GR changes its structure to release HSP90, allowing the VND7–VP16–GR protein to localize to the nucleus. This activates the transcriptional activity of VND7. **(B)** Screening of known cellular stress inducers to isolate inhibitors of xylem vessel cell differentiation. Treatment with only 10 nM DEX bleaches the *VND7–VP16–GR* seedlings because of the progression of programmed cell death. In the first screening, we checked the inhibitory activity of bleaching and obtained 12 inhibitors as positive chemicals. Subsequently, these 12 chemicals were tested for inhibitory activity of SCW deposition by confocal microscopy (second screening).

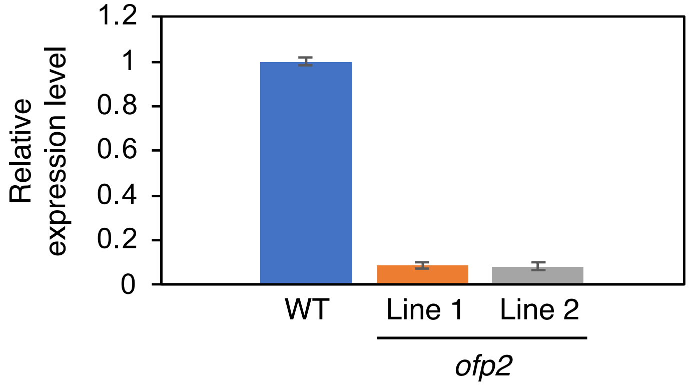

Supplementary Figure S2. The *OFP2* gene expression in two individual mutants of *ofp2*. The *OFP2* mRNA level was quantified by reverse transcription quantitative PCR. Results are shown relative to the wild type as 1.0 (means ± SD, $n = 3$).

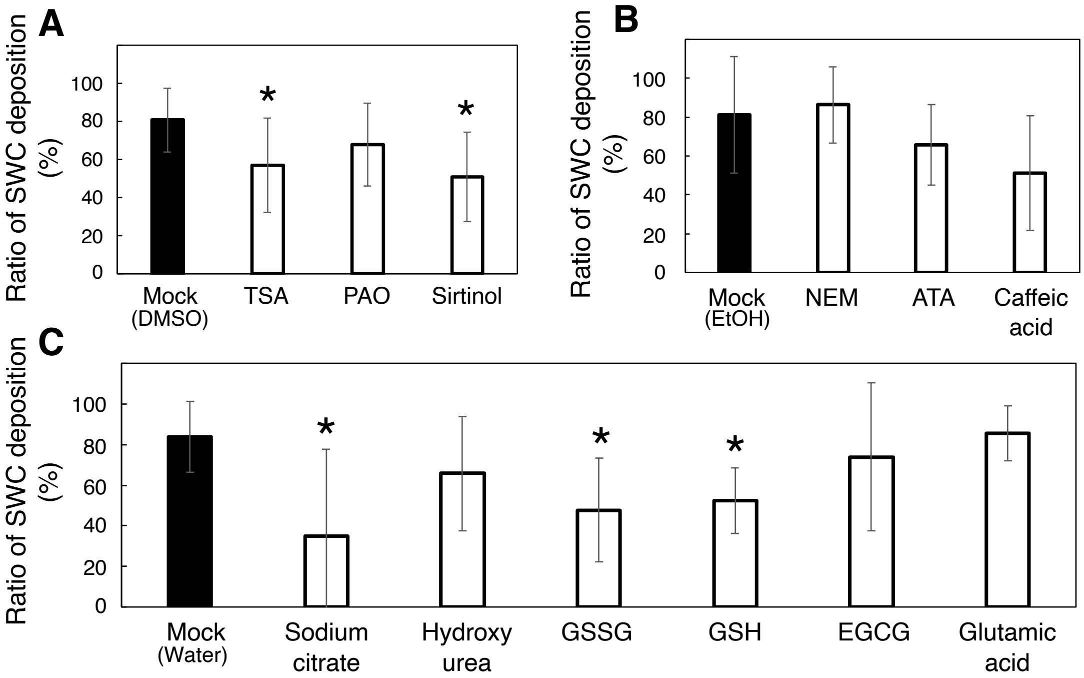

Supplementary Figure S3. Effects of selected cellular stress inducers on secondary cell wall (SCW) deposition in *VND7–VP16–GR* seedlings. Seven-day-old *VND7–VP16–GR* seedlings were treated with 10 nM dexamethasone (DEX) and either 5 μM trichostatin A (TSA), 1 μM phenylarsine oxide (PAO), 10 μM sirtinol **(A)**, 20 μM N-ethylmaleimide (NEM), 50 μM aurintricarboxylic acid (ATA), 1 mM caffeic acid **(B)**, 50 mM sodium citrate, 1 mM hydroxy urea, 3 mM oxidized glutathione (GSSG, glutathione-S-S-glutathione), 3 mM reduced glutathione (GSH, glutathione-SH), 1 μM epigallocatechin gallate (EGCG), or 1 mM glutamic acid **(C)**. Ectopic SCWs in cotyledons were observed with a confocal microscope after propidium iodide staining, and the SCW-positive regions were measured in each cotyledon. The relative proportions of SCW-positive cell regions were calculated manually using an image analysis performed in ImageJ. Results are shown as means ± SD ($n > 10$). Asterisks indicate statistically significant differences compared with the mock control (Student's *t*-test, $p < 0.05$).

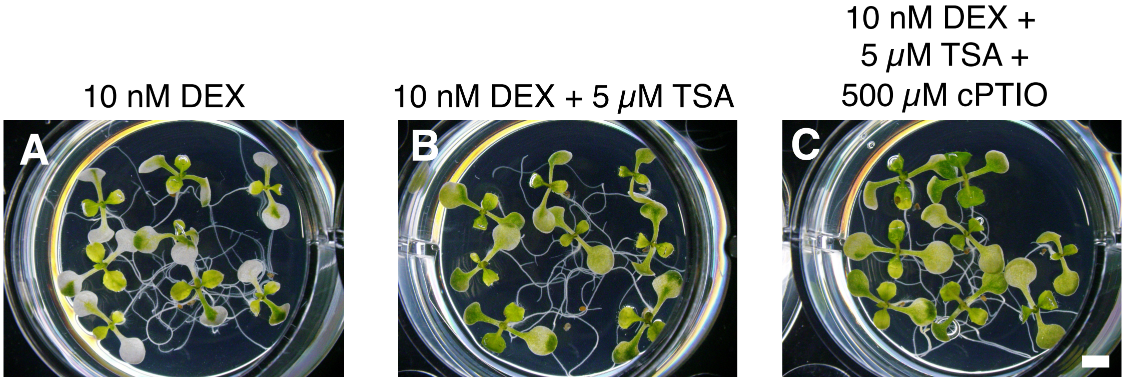

Supplementary Figure S4. Effects of nitric oxide (NO) scavenger on trichostatin A (TSA)-induced inhibition of xylem vessel cell differentiation. Seven-day-old *VND7–VP16–GR* seedlings were treated with 10 nM dexamethasone (DEX) and 5 µM TSA, with or without 500 µM 2-(4-Carboxyphenyl)-4,4,5,5-tetramethylimidazoline-1-oxyl-3-oxide (cPTIO), as an NO scavenger, for 3 d. The application of cPTIO did not affect the degree of bleaching in the *VND7–VP16–GR* seedlings. Bar, 1 mm.

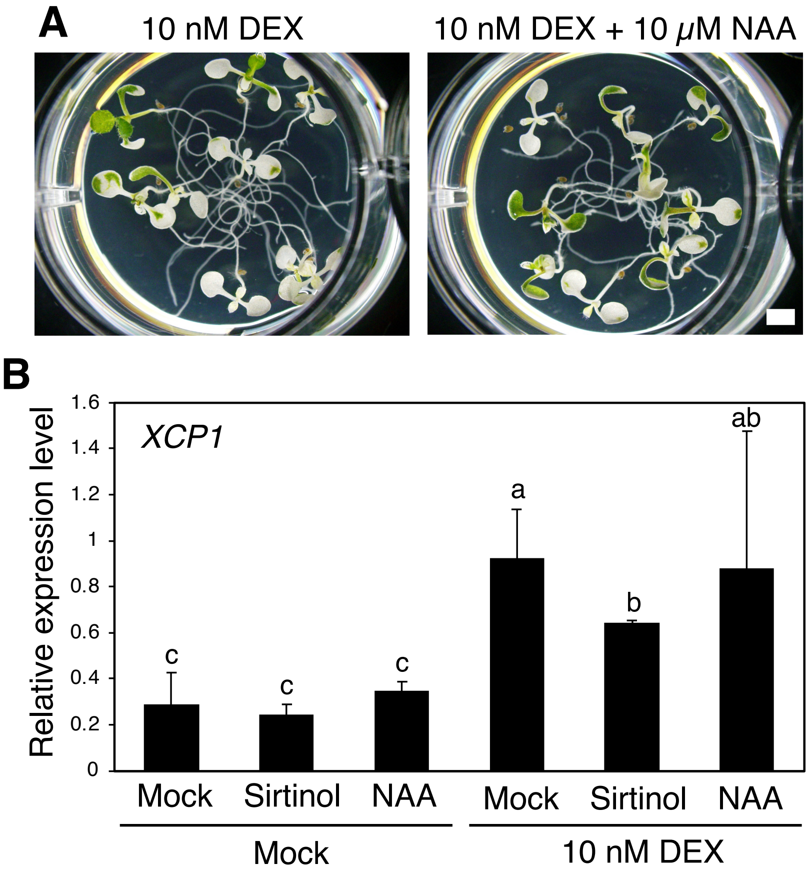

Supplementary Figure S5. Effects of auxin on xylem vessel cell differentiation in *VND7–VP16–GR*. Seven-day-old *VND7–VP16–GR* seedlings were treated with 10 nM dexamethasone (DEX) with or without 10 μM 1-naphthaleneacetic acid (NAA) for 3 d. The application of NAA did not affect the degree of bleaching in the *VND7–VP16–GR* seedlings **(A)** and the induction of *XCP1* gene, a typical direct target gene of VND7, after 24 h of DEX treatment **(B)**. Bar, 1 mm.

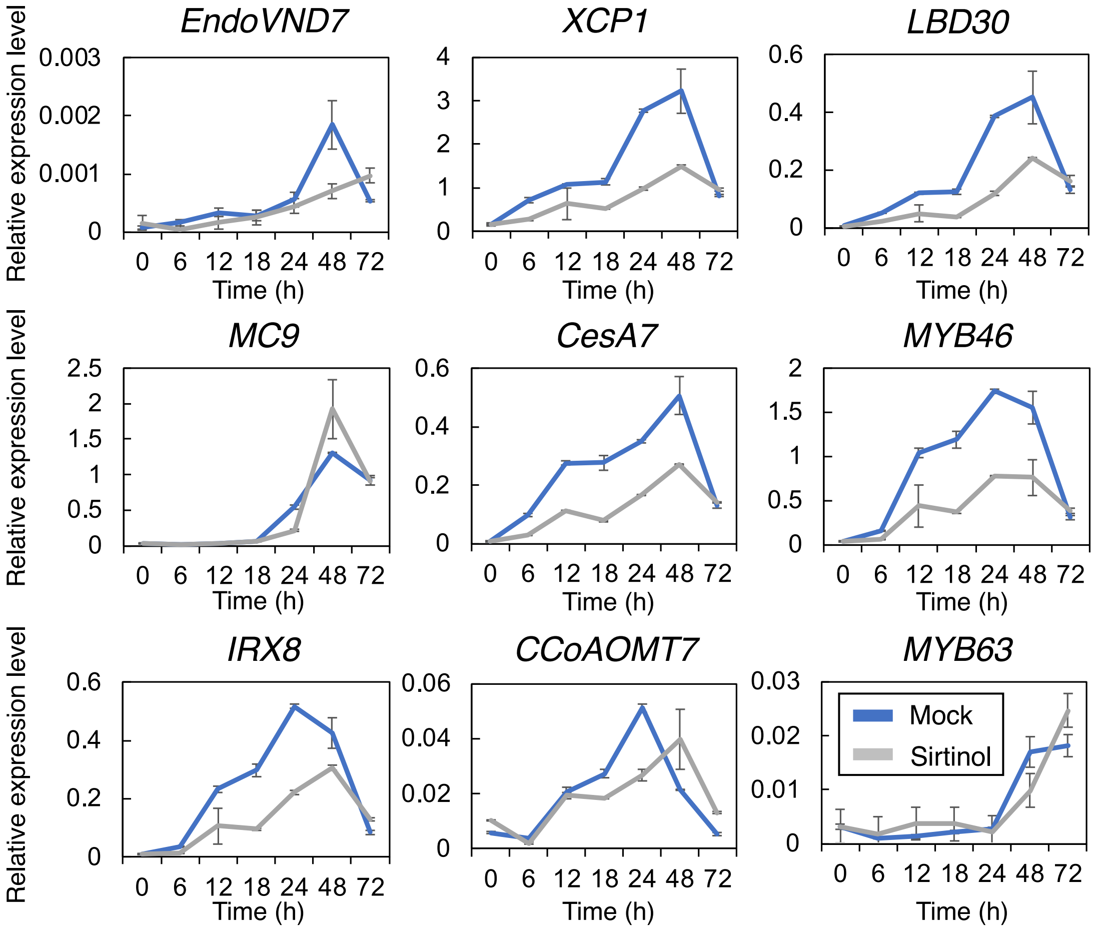

Supplementary Figure S6. Reverse transcription quantitative PCR analysis of the genes downstream of VND7 in *VND7–VP16–GR* treated with dexamethasone (DEX) and sirtinol. Seven-day-old *VND7–VP16–GR* seedlings were treated with 10 nM DEX and 10 μM sirtinol, and sampled after 0, 6, 12, 18, 24, 48, and 72 h. The expression levels of the genes downstream of VND7 were normalized to the expression level of the internal control *UBIQUITIN10*. Results are shown as means ± SD (*n* = 3).

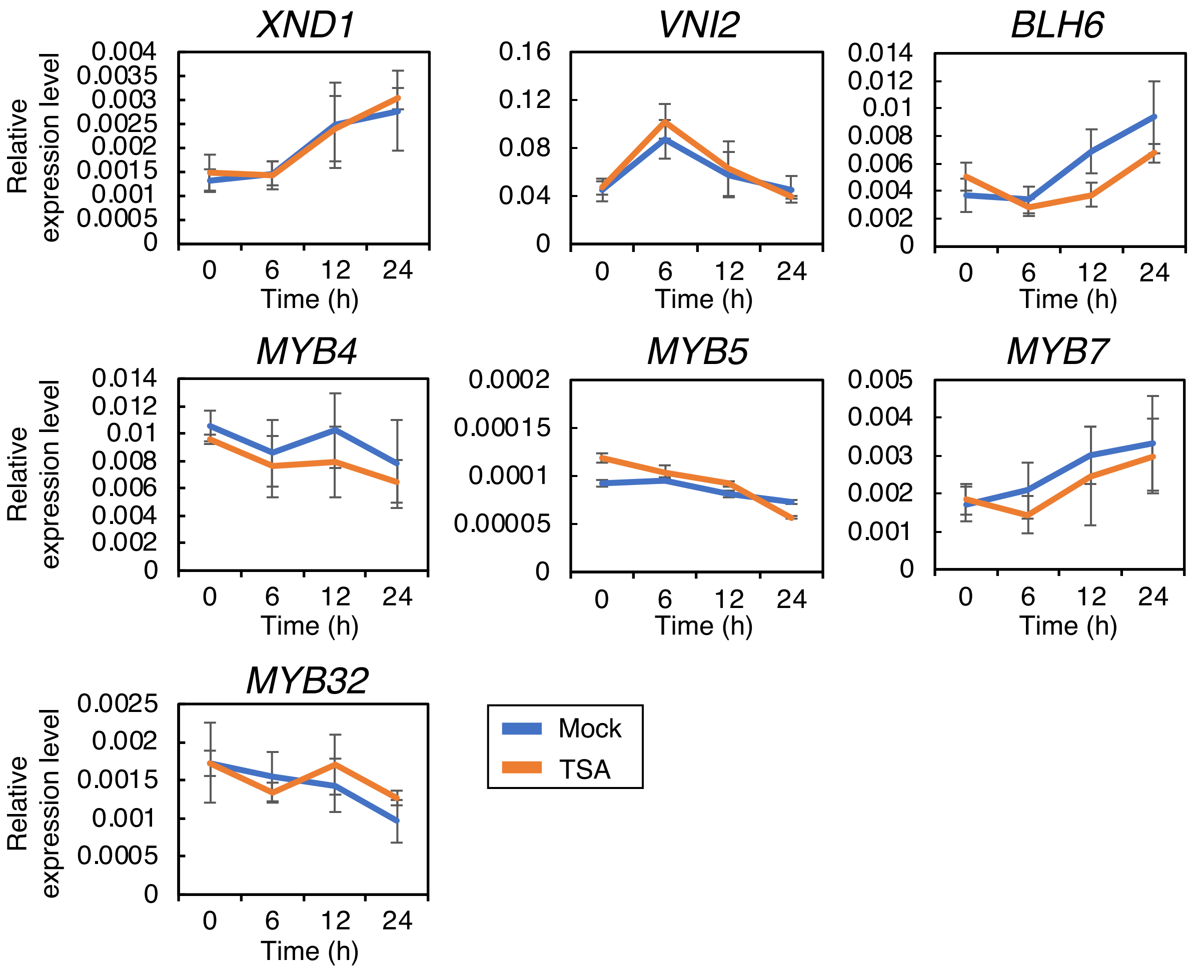

Supplementary Figure S7. Reverse transcription quantitative PCR analysis of genes for transcription factors known to negatively regulate xylem vessel cell differentiation. Seven-day-old *VND7–VP16–GR* seedlings were treated with 10 nM DEX and 5 µM trichostatin A (TSA), and sampled after 0, 6, 12, 18, and 24 h. The expression levels of the genes downstream of VND7 were normalized to the expression level of the internal control *UBIQUITIN10*. Results are shown as means ± SD (*n* = 3).

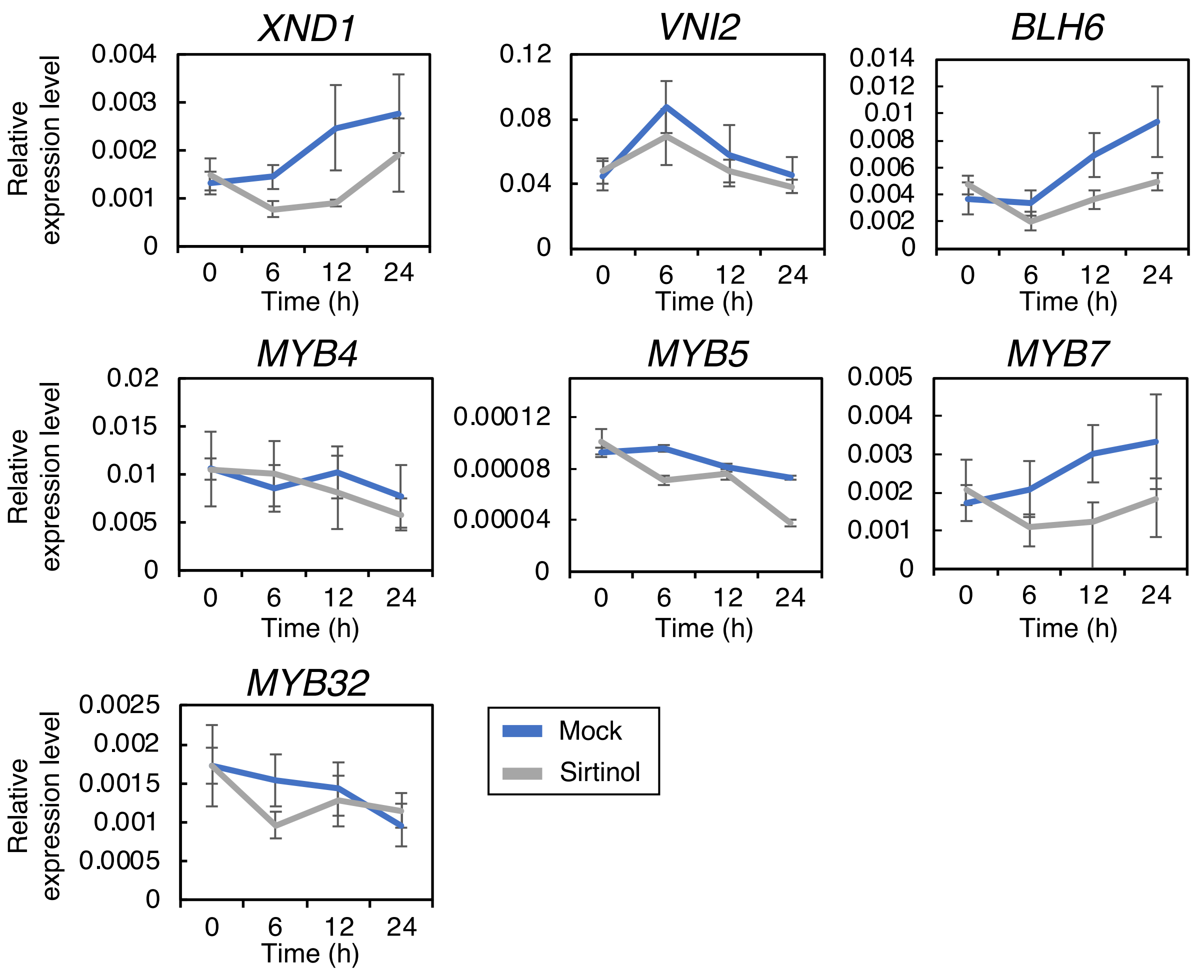

Supplementary Figure S8. Reverse transcription quantitative PCR analysis of the genes for known transcription factors negatively regulating xylem vessel cell differentiation. Seven-day-old *VND7–VP16–GR* seedlings were treated with 10 nM dexamethasone (DEX) and 10 µM sirtinol, and sampled after 0, 6, 12, 18, and 24 h. The expression levels of the genes downstream of VND7 were normalized to the expression level of the internal control *UBIQUITIN10*. Results are shown as means ± SD (n = 3).

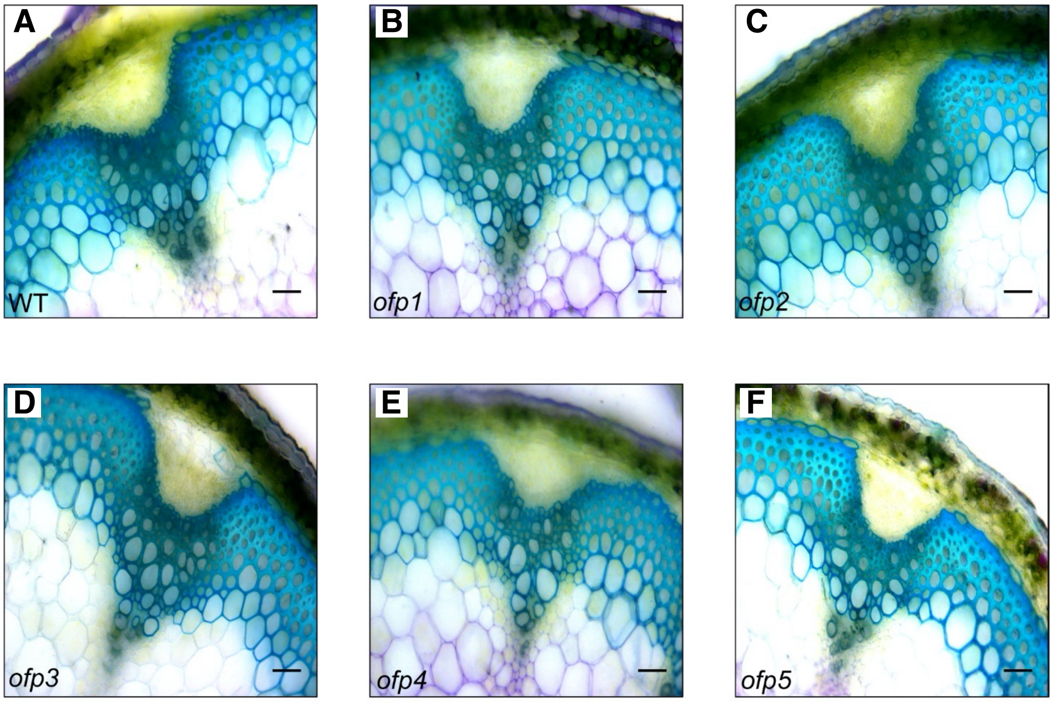

Supplementary Figure S9. Cross-sections of stem vascular bundles in wild type (WT) and *ofp* single mutants. Stem sections from the base of 8-week-old Arabidopsis plants of the wild type (WT) **(A)**, *ofp1* **(B)**, *ofp2* **(C)**, *ofp3* **(D)**, *ofp4* **(E)**, and *ofp5* **(F)** stained with toluidine blue. A single representative vascular bundle is shown from each mutant. No obvious differences are shown in xylem or interfascicular fiber morphology between *ofp* single mutants and the wild type. Bars = 20 μm.

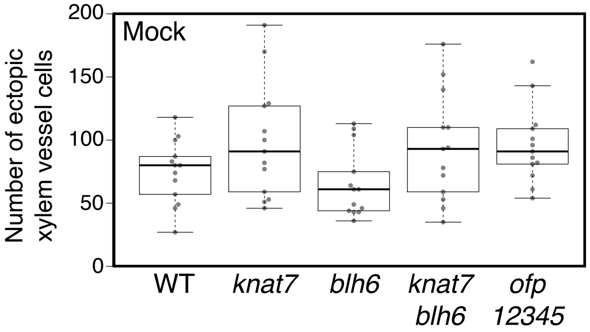

Supplementary Figure S10. Box plot of the number of ectopic xylem vessel cells per cotyledon in the KDB system (related to Figure 6). Excised cotyledons from seven-day-old wild-type, *knat7-1*, *blh6-1*, *knat7 blh6*, and *ofp1 ofp2 opf3 opf4 opf5* (*ofp12345*) seedlings were treated with phytohormones to induce ectopic xylem vessel cell differentiation. The number of ectopic xylem vessel cells under the mock treatment is shown (n = 13). In box plots, center lines show the medians; box limits indicate the 25th and 75th percentiles; whiskers extend 1.5 times the interquartile range from the 25th and 75th percentiles, outliers are represented by dots. No statistically significant differences were found among the lines.

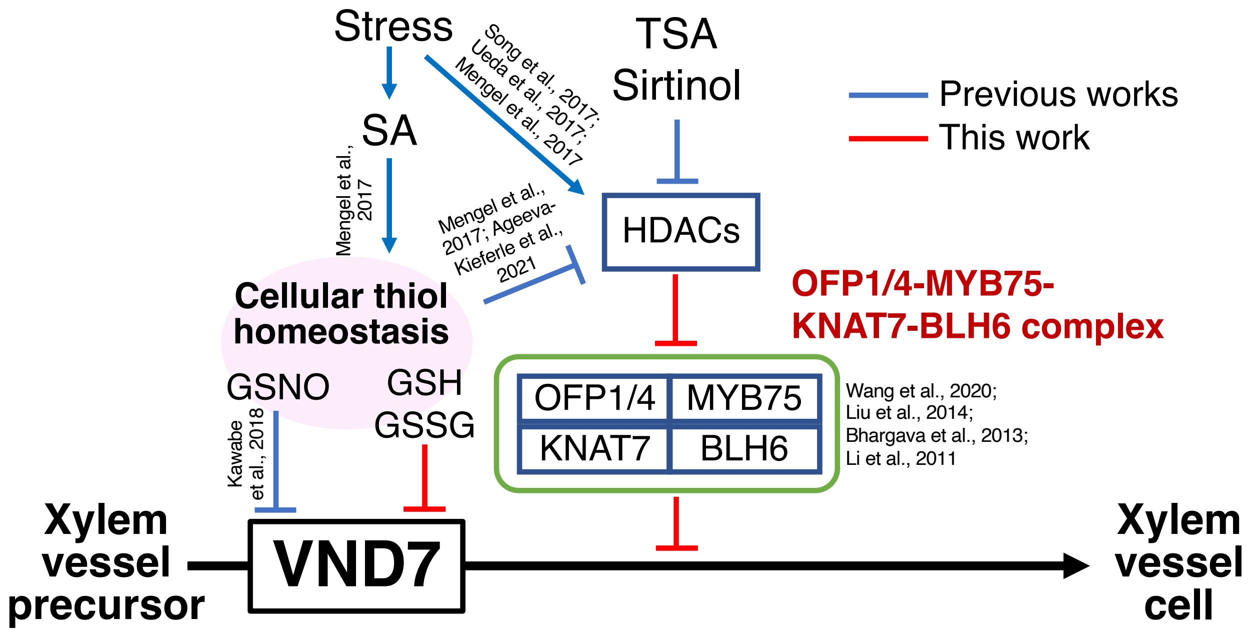

Supplementary Figure S11. Proposed role of HDAC in xylem vessel cell differentiation. Based on the results, we propose that histone deacetylation regulates the OFP1/4–MYB75–KNAT7–BLH6 transcriptional repression complex during xylem vessel cell differentiation. It is widely accepted that histone deacetylase (HDAC)-mediated regulation of histone deacetylation is a key step for relaying environmental signals to gene expression. Therefore, HDACs could control xylem vessel cell differentiation according to environmental conditions through the transcriptional repression complex.

1.2 Supplementary Table

Supplementary Table 1. Cellular stress inducers used in this study.

Chemical	Activity	Solvent	Standard concentration	Supplyer
cPTIO	NO scavenger	Water	500 mM	CAYMAN
Dithiothreitol	Reducing agent, inducer of Endoplasmic reticulum stress	Water	2 mM	nacalai tesque
Potassium nitrate	Inhibitor of plasma membrane ATPase	Water	250 mm	nacalai tesque
Chlorpromazine, Hydrochloride	Inhibitor of NO synthethase, Antagonist of Calmodulin	Water	50 mM	nacalai tesque
oxidized glutathione (GSSG, Glutathione-S-S-Glutathione)	Disturbs thiol homeostasis	Water	3 mM	WAKO
reduced glutathione (GSH, Glutathione-SH)	Disturbs thiol homeostasis	Water	3 mM	nacalai tesque
Caffeic Acid	Glutathione S-transferase inhibitor	EtOH	100 mM	Tokyo Chemical Industry
Sodium citrate	Reducing agent, Inhibitor of DNase	Water	50 mM	nacalai tesque
NG-Nitro-L-arginine Methyl Ester, Hydrochloride	Inhibitor of NO synthethase	Water	10 mM	nacalai tesque
Okadaic acid	Inhibitor of protein kinase	DMSO	1 nM	WAKO
U0126	Inhibitor of MAPK	DMSO	10 uM	CAYMAN
Staurosporine	Inhibitor of kinase	DMSO	1 uM	WAKO
Ku55933 (IATM)	competitive ATM kinase inhibitor	DMSO	10 µM	Abcam
Indomethacin	Inhibitor of phospholipase and cyclooxygenase	EtOH	10 uM	nacalai tesque
PD98059	MEK inhibitor	DMSO	10 uM	CAYMAN
N-ethylmaleimide	cysteine protease inhibitor	EtOH	200 mM	Tokyo Chemical Industry
Verapamil	Calcium channel blocker	DMSO	10 uM	Tokyo Chemical Industry
Diethyl-stilbestrol	Inhibitor of H+/K+-exchanging ATPase and calcium channel	EtOH	100 uM	Tokyo Chemical Industry
Sodium vanadate	Inhibitor of alkaline phosphatase and (Na,K)-ATPase	Water	100 µM	KANTO KAGAKU
Tunicamycin	Protein glycosylation inhibitor	DMSO	5 µg/mL	WAKO
N,N'-dicyclohexyl-carbodiimide	H+-ATPase inhibitor	EtOH	5 µM	Tokyo Chemical Industry
Hydroxyurea	Ribonucleotide reductase inhibitor	Water	1 mM	WAKO
Zeocin	Inhibitor of DNA base excision repair pathway	Water	10µM	Invivogen
Aurintricarboxylic acid	Inhibitor of Dnase	EtOH	50 uM	Sigma
W-7 Hydrochloride	calmodulin antagonist	DMSO	50 uM	CAYMAN
sodium butyrate	HDAC inhibitor	Water	100 mM	Tokyo Chemical Industry
5-aza-2-deoxycytidine	Inhibitor of DNA methylation	EtOH	20 mg/L	WAKO
Trichostatin A	HDAC inhibitor	DMSO	0.5 µM	CAYMAN
Anacardic acid	Inhibitor of histone acetyltranferases	DMSO	20 uM	CAYMAN
Curcumin	HDAC inhibitor	DMSO	40 uM	Tokyo Chemical Industry
Sirtinol	HDAC inhibitor	DMSO	10 uM	Santa Cruz Biotechnology
Epigallocatechin gallate	Inhibitor of telomerase and DNA methyltransferase	Water	500 uM	Tokyo Chemical Industry
MG132	Proteasome inhibitor	DMSO	50 uM	Peptide Institute. Inc.
E64	Cys peptidase inhibitor	Water	5 uM	Sigma
Wortmannin	PI3 kinase inhibitor	DMSO	10 uM	AdipoGen
Phenylarsine oxide	PI4 kinase inhibitor	DMSO	10 uM	Santa Cruz Biotechnology
Pladinoride B	pre-mRNA splicing inhibitor	DMSO	1 uM	CAYMAN
5-fluorouracil	Inhibitor of RNA biosynthesis	DMSO	45 mM	WAKO
Silver Thiosulfate(Silver Nitrate + Sodium Thiosulfate)	Inhibitor of ethylene signaling	Water	6 uM	Sigma
Streptomycin	Inhibitor of protein synthesis of bacteria and organella	Water	200 mg/L	nacalai tesque
3-(3,4-dichlorophenyl)-1,1-dimethylurea (DCMU)	Inhibitor of phytosynthesis system II	EtOH	10 m M	WAKO
8-Br-cAMP	Activator of cyclic-AMP-dependent protein kinase	Water	10 mM	CAYMAN
ODQ(1H-[1,2,4]Oxadiazolo[4,3-a]quinoxalin-1-one)	Inhibitor of no-sensitive guanylyl cyclase	DMSO	4µ M	CAYMAN
EDTA2Na	Chelating agent	Water	10 mM	WAKO
o-phenanthroline	Inhibitor of matrix metalloproteinase (MMP)	Water	2 mM	nacalai tesque
Cdk4/6 Inhibitor IV	Cdk4/6 inhibitor	DMSO	1 mM	CAYMAN
Trimethoprim	Inhibitor of tetrahydrofolic acid synthesis	DMSO	50 mM	nacalai tesque
Glyphosate	Inhibitor of Shikimate pathway	Water	0.5 mM	Sigma
1-Triacontanol	Plant growth regulator	Chloroform	0.5 uM	CAYMAN
Glutamate	Signal mediator	Water	1 mM	nacalai tesque

Supplementary Table 2. Primers used in this study.

Primers for qRT-PCR	
**Primer Name**	**Sequence (5'-3')**
OFP1-L	ATGGGTAATAACTATCGGTTTA
OFP1-R	GCTATTTGGTTGGCTCTGAAGATTCT
OFP2-L	AGAGCAAACAAGATGTTCTA
OFP2-R	TTTGTAAGTTGAAGCCAGAT
OFP3-L	ATGAAACAGAAAATGGGGAC
OFP3-R	TTGGGAGAAGAAAGATGGTG
OFP4-L	ATGAGGAACTATAAGTTAAGA
OFP4-R	TATGGAGTAAAGAGGAAGAGA
OFP5-L	GATGGAGGAATGGAGAACGA
OFP5-R	TTATGCACCTGCTGGAACAC
MC9-qRT F	GTGCCATGAAGAACAAGCAA
MC9-qRT R	AGAAAGGAACGTCGCGTCT
CCoAOMT7-qRT F	ggaaactaagacttcagtcatctgag
CCoAOMT7-qRT R	gagctcttcacttttcagcaatc
MYB63-qRT F	aacagctcaggctcaagagcaac
MYB63-qRT R	atgtatcatgagctcgtagttctt
BLH6-qRT F	caagacaaacgggcttgag
BLH6-qRT R	ttcctccttgtatatctcctcca
SRT1 qRT F	AGACCTGAAATGGAAGCTGG
SRT1 qRT R	CTATGCCTTGGTTTCTTCTGCC
SRT2 qRT F	TCATGAGGCTGGTGCTATGAC
SRT2 qRT R	CTAGAGAGCTGGGACACTGAG
MYB75 qRT R	AAGCCTATGAAGGCGAAGAA
MYB75 qRT L	TGGCACCAAGTTCCTGTAAG
OFP1 qRT R	gaatcttcagagccaaccaaa
OFP1 qRT L	gtagggactgcgtggtgttt
KNAT7 qRT F	CGAGAATCGAAGATGTAAGAG
KNAT7 qRT R	GTGTTTGCGCTTGGACTTCAA
XND1-qRT F	attacgatccttgggaccttc
XND1-qRT R	tgcttgtcactctctcttgtgtc
OFP4 qRT F	catttcatcaaagccatgattct
OFP4 qRT R	cagaagaaatgactttcaagttgg
MYB4 qRT F	actatctccggcctgacctt
MYB4 qRT R	cggcaataagcgaccatt
MYB7 qRT F	tgttggcgatctcttcctaga
MYB7 qRT R	aagacggcagcttttaccg
MYB32 qRT F	cttgtcaagattctctcttttggtc
MYB32 qRT R	aattctctcgtccccgaaat
MYB5 qRT F	aattacgtcggacgaggaag
MYB5 qRT R	cttcccgcgatcaatgac
XCP1-for1	TTGACCCATGAAGAGTTCAAAGGAAGA
XCP1-rev1	GAAAGCGAACTCAGATTCCCTGTTG
MYB046-for2	GAATGTGAAGAAGGTGATTGGTACA
MYB046-rev4	CGAAGGAACCTCAGTGTTCATCA
LBD30-for4	CTATCTACGGCTGCGTCTCTCACATCGT
LBD30-rev7	TAGAGATCCTGAAGATGACACCGGAAC
Ubq10-for1	AACTTTGGTGGTTTGTGTTTTGG
Ubq10-rev1	TCGACTTGTCATTAGAAAGAAAGAGATAA
CesA7-RT-for1	ATGGGTAGACAGAACAGAACACCAA
CesA7-RT-rev1	CTTCAGCAGTTGATGCCACACTT
IRX8-RT-for1	TCAAGAGCTGTCACATTAGAGCAT
IRX8-RT-rev1	ATGATCCGGTAGAGAAGTGAAAAC
endo_VND7_RT_F	aatacgtttataggatcatcgtgg
endo_VND7_RT_R	TTTGATAGTACCGCCTTGTCTCTAC
exo_VND7_RT_F	cttggatgcttccctgactc
exo_VND7_RT_R	gtcctcgccgtctaagtgg

**Primers for genotyping of *opf* mutants**		
Mutant	**Primer Name**	**Sequence (5'-3')**
ofp1	SM_3_21689-L	ATGGGTAATAACTATCGGTTTAAG
	SM_3_21689-R	TTATTTGGAATGGGGTGGTGGAA
	Tran-element	TACGAATAAGAGCGTCCATTTTAGAGTGA
ofp2	SALK_122550-L	ACCAAATTCAAAGAAGCATCG
	SALK_122550-R	TGGTGAGTTATGGTGAGGAGG
	LBb1.3	ATTTTGCCGATTTCGGAAC
ofp3	GABI_167F01-L	CAGAAAATGGGGACTCACAAG
	GABI_167F01-R	TGACTTTGAGAAAGAGGACGG
	GK T-DNA	ATATTGACCATCATACTCATTGC
ofp4	SALK_022396-L	ATGAGGAACTATAAGTTAAGATTG
	SALK_022396-R	CTACTTCGATGCAAATGTAGAG
	LBb1.3	ATTTTGCCGATTTCGGAAC
ofp5	SALK_203823-L	GACAACATCTTCATCTCCCTCC
	SALK_203823-R	ATTATGCACCTGCTGGAACAC
	LBb1.3	ATTTTGCCGATTTCGGAAC
